# Remote data collection during COVID-19 restrictions: an example from a refugee and asylum-seeker participant group in the UK

**DOI:** 10.1186/s13063-021-05058-2

**Published:** 2021-02-05

**Authors:** Lauren Walker, Della Bailey, Rachel Churchill, Emily Peckham

**Affiliations:** 1 Mental Health and Addiction Research Group, University of York, Heslington, YO10 5DD UK; 2 York Mental Health Research Group, University of York, Heslington, YO10 5DD UK; 3 Centre for Reviews and Dissemination, University of York, Heslington, YO10 5DD UK

**Keywords:** Remote data collection, COVID-19, Lockdown, Refugee, Asylum seeker, Arabic, Digital divide, Risk management

## Abstract

**Abstract:**

This article describes how one trial site of the Refugee Emergency: Defining and Implementing Novel Evidence-based psychosocial interventions (RE-DEFINE) study, designed to evaluate a Self Help+ intervention with Arabic-speaking refugees and asylum seekers currently living in the UK and experiencing stress, was adapted to accommodate social distancing rules and working from home during the COVID-19 restrictions. Digital divide, risk and safety management, acceptability of remote data collection and practical considerations are described. The adaptions to methods have practical implications for researchers looking for more flexible approaches in response to continuing restrictions resulting from COVID-19, and the authors believe that others could adopt such an approach. The need for a further acceptability study focusing on human and economic costs and benefits of telephone and video as an alternative to face-to-face data collection is indicated.

**Trials Registration:**

Refugee Emergency - Defining and Implementing Novel Evidence-based psychosocial interventions RE-DEFINE. (Trials registration numbers NCT03571347, NCT03587896) 10.1136/bmjopen-2019-030259 (2019)

Data collection for a research intervention, RE-DEFINE and Self Help+ trial (trials registration numbers NCT03571347, NCT03587896) [1], involving a refugee and asylum-seeker population in the UK proved challenging but also presented a learning experience during the COVID-19 lockdown. The research upon which this paper is based was international, and the response to the pandemic varied according to the country and its specific restrictions. This article discusses the trial from the perspective and experience of researchers working on RE-DEFINE in England (trial website http://re-defineproject.eu/re-define-project/).

Follow-up research questionnaires taking approximately 60–90 min with a research population of Arabic-speaking refugees and asylum seekers currently living in the UK that were conducted by a researcher and cultural mediator (individuals with fluent spoken and written English and Arabic and experience of supporting others, employed to work with researchers on RE-DEFINE interpreting questionnaires and answers between researcher and participant) were moved from in-person meetings to three-way phone or video calls with little to no disruption to expected follow-up dates and participant retention. This article details the steps taken to achieve this.

Participants (who were already enrolled in RE-DEFINE), Arabic-speaking refugees and asylum seekers currently living in the UK and experiencing stress (score of 3+ on the 12-item General Heath Questionnaire (GHQ-12)) but not currently meeting the criteria for a diagnosable mental health condition (according to the Mini-International Neuropsychiatric Interview), were contacted by phone by cultural mediators who explained the move to remote follow-up questionnaires instead of in-person meetings and booked appointments. Questionnaires could not be moved to an online format due to the fact that the PROM (Mini-International Neuropsychiatric Interview) was in English only (no Arabic translation); there were questions about suicide and a risk protocol to follow as well as the fact that some participants could not read or write. The role of cultural mediators and the telephone or video call format was vital. During a time when daily routines and diaries were interrupted for many people, participants were sent appointment reminders by text. Reminders of appointment dates and times were appreciated by participants and saved time for the researcher and mediators.

Researcher and mediator flexibility with participants was vital. Occasionally, participants did not answer the phone at the agreed appointment time or were late. Internet connectivity and stop/start call experiences were not always optimal, and choice of voice or video call preference and choice of call platform were essential. Three-way WhatsApp audio call was the preference for the vast majority (approximately 90%) of participants. Participant preference for audio calls meant that the researcher had to rely on cues from cultural mediators slightly more than usual as visual, and non-verbal cues are important when participants and researchers do not share a common spoken language. The research population were generally familiar and comfortable with multi-way calls and video calls due to many having family and friends in other parts of the world.

Insecure platforms (those known to be insecure when COVID-19 restrictions began due to, for example, ‘video bombing’) were not used in this study. The research team already had personal mobile phone numbers for participants and had met all of the participants before. The cultural mediators booked appointments with participants before making the research follow-up calls, and names and addresses were checked for identification.

Challenges encountered by the researcher included concerns about participant privacy (for participants in lockdown with family members, friends or in shared accommodation) and privacy for researchers and mediators working from home. However, to date, there have been no concerns of this nature for either the researcher or cultural mediators. Paper questionnaires were anonymised and locked away by the researcher, participant data was stored on a computer in locked/encrypted spreadsheets and no recordings were made of conversations with participants. As this was not an NHS study, appropriate checks were made and amendments were not needed. The risk protocol was adjusted appropriately in consultation with the site PI and clinical colleagues.

Access to resources to pay for services (WiFi and data), physical access to technology and necessary skills to take part in calls is a digital divide issue for refugee migrant groups (Alam and Imran [2]). However, in socioeconomically disadvantaged groups, motivations for self-efficacy, knowledge and cultural capital led to intended and continued information and communications technology (ICT) use (Po-An Hsieh et al. [3], pp. 213–417). Cultural mediators and participants have informally referred to the above motivations as high in this population. Demonstrations of participant commitment and effort to participate in challenging circumstances included participants borrowing data from a flat-mate in order to take part, taking a walk to answer questionnaires for privacy and splitting the follow-up into two separate calls.

Face-to-face meetings with participants allow for the researcher to observe non-verbal cues such as body language alongside verbal communication when gathering information, which is important when asking sensitive questions around, for example, risk or suicide prevention. Many of the participants are from backgrounds where talking about suicide is difficult for reasons of culture and faith, and in these circumstances, researchers can sometimes become aware of a mismatch between body language and verbal responses to risk questions. This is more difficult to observe on a video screen and impossible by phone.

Before making the first follow-up call, the risk protocol was adapted to remote working by researchers and clinicians working on the trial. All participants were asked for their current address and the address they were taking the call from at the beginning of the phone call, before any research questions were asked. The researcher had two phones available for making any calls to risk assessors or health professionals, enabling them to keep the participant on the other line with the cultural mediator. Knowledge of the address that the participant was participating in the call from meant that they could be kept safe in a situation where risk was be identified that meant an ambulance was required.

The positive working relationship already built between the researcher and cultural mediators served to facilitate a smooth transition to remote working. Follow-up questionnaires being carried out at 6-month and 12-month follow-up points also meant that participants were already familiar with the process, content (questionnaires) and the researcher and mediator.

The researcher observed participant resilience, flexibility and creativity in a large number of participants, but also difficulties with experiencing a further trauma for some in a refugee and asylum-seeker population already selected for their experiences of stress (score of 3+ on the GHQ-12).

Participants in this trial received a voucher to thank them for their participation. In this potentially vulnerable population, it was important that despite lockdown, vouchers were sent by post in a timely manner in order to honour the commitment of the research participants.

The process of data collection by phone was completed in a similar and possibly shorter time (average 60–90 min by phone as compared to 90–120 min in person). The shorter time frame is possibly due to less informal chat with participants. The reduction in travel time ranged from 30 min locally to several hours for a researcher and mediator travelling to a different area of the country, and travel expenses were eliminated by remote follow-up methods. One of the mediators working on the study commented that the work was quicker and simpler by phone. The method is familiar, convenient and acceptable to participants (from informal feedback), and time and cost-saving for researchers, potentially allowing researchers to cover wider geographical areas for follow-up phases of similar studies.

We have seen the 6-month follow-up retention rate to be almost identical in those followed up before and after the pandemic restrictions. The retention rate drops at 12 months which is to be expected, and as this is a transient population, it is not possible to speculate any specific effects of moving to virtual/ remote follow-up methods.

The fact that the researcher and cultural mediators were already working remotely and flexibly on this trial meant that the changes possibly had a lesser impact than for others moving to working from home and remote data collection during COVID-19 lockdown.

In conclusion, suggestions for a future approach based on our learning during the pandemic points to a potential mix of in-person and remote (telephone or video call) data collection methods going forward, considering participant preferences and research findings on the costs and benefits of remote data collection. The authors believe that others could adopt such an approach and that this paper will provide insight into what they need to know. In this study, the researcher and mediators had already met the participants at baseline, and so, questionnaires by phone during the restrictions were follow-ups only. In the future, it may be possible to follow this model of meeting face to face for baseline measures and following up by phone, saving time and expense on unnecessary travel.

The experience of this process suggests a possible basis for a wider acceptability study evaluating the human and economic costs and benefits of telephone and video data collection as an alternative to face-to-face data methods.

## Data Availability

N/A.

## Abbreviations

COVID-19: Coronavirus disease 2019
RE-DEFINE: Refugee Emergency: DEFining and Implementing Novel Evidence-based psychosocial interventions
GHQ-12: 12-item General Health Questionnaire
ICT: Information and communications technology
